# High Intake of Sodium Chloride for 28 Days Causes No Effect on Serum FGF23 Concentrations in Cats

**DOI:** 10.3390/ani12223195

**Published:** 2022-11-18

**Authors:** Carla Steffen, Ellen Kienzle, Britta Dobenecker

**Affiliations:** Chair of Animal Nutrition and Dietetics, Department of Animal Sciences, Ludwig-Maximilians-Universität München, Schoenleutnerstr. 8, D-85764 Oberschleissheim, Germany

**Keywords:** FGF23, phosphate, chronic kidney disease, renal health

## Abstract

**Simple Summary:**

Two important functions of the hormone fibroblast growth factor-23 (FGF23) are the regulation of phosphorus homeostasis and sodium retention. Dietary sodium chloride is known to affect FGF23 level and blood pressure in humans and mice. Furthermore, increased serum FGF23 concentrations are associated with chronic kidney disease, which is the most common cause of death in aging cats. It is therefore of interest to investigate the potential influence of high sodium chloride intake on FGF23. In contrast to findings in mice and humans, high sodium chloride supply did not affect serum FGF23 concentrations in cats. Apparent digestibility of phosphorus, on the other hand, was significantly increased by adding sodium chloride to the diet. Therefore, high dietary sodium chloride concentrations may increase the phosphorus burden on the body. This is especially true when phosphate originates from highly available sources, such as inorganic phosphates with high solubility.

**Abstract:**

Background: FGF23 is an acknowledged parameter to assess kidney health. As chronic kidney failure is one of the most common diseases in aging cats, dietary influences on renal health warrant investigation. The purpose of this study was therefore to investigate potential correlations between dietary sodium chloride and FGF23. Methods: In a total of two trials, 11 cats were included. In the first trial, the cats were fed a complete and balanced control diet; in the second trial, sodium chloride was added (8 g/kg/DM)). Blood, urinary, feed, and faecal samples were analysed for major minerals. FGF23 and creatinine were measured in blood and urine samples. Results: Serum phosphate and FGF23 were unaffected by high sodium chloride intake, thus showing no correlation between serum FGF23 and sodium concentrations. Apparent phosphorus digestibility was significantly increased, however, by high sodium chloride intake, whereas apparent digestibility of calcium was unaffected. The present study confirms differences in FGF23 and sodium chloride interaction in cats compared with other species. Further research regarding the correlation between sodium chloride and phosphate homeostasis is warranted.

## 1. Introduction

**Figure 1:**
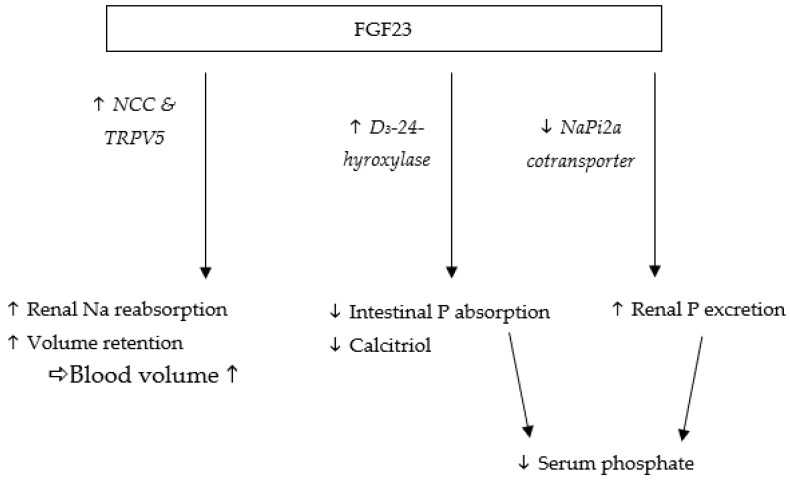
Selected physiological effects of FGF23 on sodium and phosphate metabolism, as established in humans and rodents.

Fibroblast growth factor-23 (FGF23) is one of the most important hormones for regulating the serum levels of inorganic phosphate and is produced by osteoblasts and osteocytes [1,2]. Its pivotal role as phosphatonin is mediated by FGF receptors (FGFR) in combination with their co-receptor αKlotho [3]. FGF23 reduces reabsorption of phosphate in the proximal tubules of the kidney (which is one of its target organs) by degrading the sodium-phosphate cotransporter NaPi2a [4] (Figure 1). By decreasing phosphate retention, FGF23 lowers serum phosphate levels [5]. In contrast, high phosphate intake leads to an increase in serum phosphate and FGF23 concentrations, as shown in cats [6].

As demonstrated in mice [7], FGF23 also decreases serum 1,25-dihydroxyvitamin D (vitamin D) levels, which in turn reduces intestinal absorption of calcium, and potentially of phosphate [8,9]. Hence, humans and mice deficient in αKlotho and FGF23 develop increased vitamin D levels and soft tissue calcifications, due to increased calcium absorption [10,11]. Furthermore, low vitamin D serum levels cause an activation of the renin-angiotensin-aldosterone system (RAAS) in the kidney. Therefore, FGF23 may upregulate RAAS by decreasing calcitriol, which is a potential negative regulator of this system [12]. As a response, αKlotho expression decreases, promoting even higher FGF23 serum levels, resulting in a loop mechanism [13,14]. 

The connection between FGF23 and phosphate serum levels has raised interest in investigating FGF23 as a potential marker for disturbed phosphate metabolism in chronic kidney disease (CKD) in humans [15,16], cats [17,18], and dogs [19]. Increased serum phosphate levels have been shown to be negatively correlated with life expectancy in CKD patients of various species, including humans [20,21] and cats [22,23]. Understanding the regulatory mechanisms behind phosphate metabolism is therefore crucial. IFGF23 also influences renal calcium and sodium excretion in humans [5] and mice [24]. In the distal kidney tubules, FGF23 upregulates the expression of the sodium-chloride cotransporter (NCC). FGF23 also upregulates the transient receptor potential vallinoid-5 (TRPV5) for cations and especially calcium, which increases sodium and calcium reabsorption (Figure 1). In CKD patients, serum FGF23 concentrations are often increased, causing higher sodium retention with potential effects on blood pressure [25,26,27]. In humans [28,29] and mice [30], high intake of sodium chloride has been shown to increase blood pressure, which is partially explained by an increase in intravascular volume [31]. FGF23 may therefore increase renal sodium uptake independent of the RAAS system, leading to volume expansion and increased blood pressure [24]. As a counteractive mechanism, serum FGF23 concentrations and sodium retention decrease in order to lower blood pressure [24,32,33]. Consequently, renal sodium reabsorption is reduced in cases of low FGF23 levels, as demonstrated in FGF23-knockout mice [34]. FGF23 deficiency was found to decrease sodium retention and lower blood pressure despite high sodium chloride intake [24]. Hypertension is one of the main causes of cardiovascular disease in humans [35], and exacerbates chronic illnesses, such as kidney disease [36]. Kidneys with a reduced glomerular filtration rate are particularly sensitive to fluctuations in blood pressure [37]. Increased serum sodium levels caused by high sodium chloride intake adversely affect kidney health by elevating blood pressure in humans [38,39,40]. As a result, chronic hypertension can cause vascular and glomerular injury, leading to nephrosclerosis [41]. FGF23 also directly affects the cardiac tissue. In humans and mice, increased serum FGF23 concentrations have been linked to left ventricular hypertrophy as an independent factor [42,43]. Furthermore, in human patients with RAAS blockage and on a sodium-restricted diet, an elevated serum FGF23 concentration was correlated with proteinuria [44]. Therefore, the role of FGF23 in regulating sodium excretion with RAAS is an important factor to consider in renal patients [45]. In cats, hypertension is less likely to occur in healthy individuals compared with feline CKD patients (hazard ratio = 0.2) [46]. The reported prevalence of hypertension as comorbidity in feline CKD patients ranges from 19 to 65% [46,47]. Renal failure represents the most common metabolic disease in feline patients [48,49], and with up to 80% of geriatric cats reported to develop CKD, it is considered the leading cause of death [50]. Hence, determination of the effects of high sodium chloride intake on FGF23 is important in this species, despite no effect being observed for high oral sodium chloride intake (3.1 g/1000 kcal) on blood pressure in cats [51]. This contrasted to findings in mice and humans.

A study in salt-sensitive Dahl rats found a correlation between increased serum FGF23 and phosphate concentrations, as well as a reduction in αKlotho values [52]. Considering that sodium is involved in phosphate homeostasis and as FGF23 partakes in the regulation of serum phosphate, a potential connection between dietary sodium chloride and serum FGF23 concentrations may also affect phosphate balance. Therefore, potential factors influencing FGF23 serum concentrations, such as dietary sodium chloride and phosphate intake, need to be considered, especially when formulating diets for CKD patients. Dobenecker et al. (2018) demonstrated that the source of phosphate needs to be considered: sodium phosphate (NaH_2_PO_4_ + 2H_2_O) and calcium phosphate (Ca(H_2_PO_4_) + H_2_O) added to the diet of cats led to an increase in renal phosphate excretion, although apparent phosphate digestibility did not significantly differ from control diets [53]. In dogs, the apparent digestibility of total phosphate was also not influenced by these sources [54,55]. 

This study aimed to gain further insight on serum FGF23, and the balance of major minerals and parameters of renal health in cats by the selective increase of sodium chloride supply with an otherwise balanced diet. 

## 2. Animals, Materials, and Methods

Two trials, each with a duration of 28 days (d), were conducted. Eleven healthy adult European shorthair cats (4 males, 7 females, 1–4 years of age, 2.7–4.7 kg body weight), bred and housed in the cattery of the Chair of Animal Nutrition and Dietetics, Department of Veterinary Sciences, Ludwig Maximilian University of Munich, participated in this study. Before commencing the study, a complete blood count and clinical blood chemistry, including markers of renal health (urea, creatinine, SDMA, and serum electrolytes), were evaluated for each cat to ensure proper health status. Both 28-day trial periods consisted of an adaptation phase for 18 days, followed by a digestibility trial for 10 days. During the digestibility phase, urine and faecal samples were quantitatively collected; food and water intake were measured. Each trial was completed on day 28 by taking blood samples pre-prandially (minimum 12 h after the last meal), as well as 3 h postprandially. Cats were daily examined by a veterinarian and underwent a detailed weekly general clinical exam and weighing. 

### 2.1. Housing

All cats were kept in groups of 4 to 8 animals during the 18 days of the adaption phase and were only single-housed during feeding, for a maximum of 2 h a day. During the digestibility trial, the cats were housed individually in cages (length × width × height (cm) = 120 × 60 × 53 or 90 × 80 × 75), to which they were accustomed and reintroduced to in the weeks prior to the trial. Light (natural and artificial) was available for at least 8 h a day and air temperature and humidity were adjusted by an air-conditioning system. Fresh water was provided in stainless steel bowls ad libitum, whereas the amount of feed was calculated and apportioned based on the individual energy requirements, according to historical data, to allow for maintenance of individual body weight. Each kennel had seat boards and was equipped with blankets and a litter box. 

### 2.2. Diets

In the first trial (CON), the cats were fed a complete and balanced control diet to establish basic values (Table 1). The second trial (HNaCl) aimed to investigate the influence of a high sodium chloride intake (10 times the recommended level according to FEDIAF, 0.8 g/kg DM) [56] by adding sodium chloride to diet CON (Table 1). The cats were fed two equal meals per day. Both diets contained phosphate of organic sources only, meeting the daily recommendation of phosphorus intake (FEDIAF, 2.6 g/kg DM) [56]. In order to adjust the cation–anion balance, potassium chloride was added to the HNaCl diet, doubling the potassium intake compared with CON (3 times the recommended level of potassium according to FEDIAF, 6 g/kg DM) [56].

### 2.3. Sample Collection and Storage

Total water intake (drinking water and moisture content of the food) was measured per day. The amount of evaporated water was determined by weighing an identical water bowl during the same time period and factored in. Urine was collected via a double-layer toilet system, with two plastic basins stacked on top of one another. To allow normal feline behaviour while using the litterbox, the first basin was filled with inert polyethylene beads as litter material. The fresh urine passed through slots in the bottom and accumulated in the second basin, which was prepped with a mixture of thymol and paraffin to preserve the urine until collection. Urine was collected repeatedly during the day with a maximum period in between of 12 h (overnight). Known from in-house trials, using thymol and paraffin is a reliable method to conserve the stability of urine pH over a period of at least 12 h. Additionally, to verify effectiveness of the method, the pH was measured in several fresh urine samples and the consistency of pH values in the preserved samples was repeatedly evaluated over a period of 12 h. The amount of urine was determined by weight. To collect the urine, the surface of the paraffin-thymol mixture was penetrated, and the urine was carefully extracted. Directly after sample collection, pH (WTW pH 325, calibrated before measuring) and specific weight (refractometer HRM 18, Krüss Optronic, Germany) were measured. Samples were kept refrigerated, pooled per day, and stored at −18 °C thereafter. Faeces were collected as soon as defecation was noticed, then weighed and stored at −18 °C until analysis. After the faeces samples were freeze-dried (T 22 K-E-6, Piatkowsky, Munich, Germany), daily samples were pooled, ground, and thoroughly mixed. Blood for serum and citrate plasma was drawn at fixed time points: the first sample was taken preprandially (starting at 7 a.m., pre; at least 12 h fasted) from either the vena saphena medialis or vena cephalica ntebrachia. Afterward, the cats received their morning meal. The second blood sample was taken 3 h postprandially (ppr; after the morning meal). After allowing the blood to clot for ~30 min and ~2 h (FGF23), respectively, samples were centrifuged for 15 min at 2000 rpm (FGF23) and 10 min at 3000 rpm for the remaining parameters. Until analysis, all samples were stored at −80 °C. Before analysis, feed and faecal samples underwent wet digestion with 65% HNO_3_ in a microwave system. Urine samples were gently thawed and an aliquot of each 24 h sample was pooled after stirring and used for analysis. 

### 2.4. Laboratory Analyses

Blood, urine, feed, and faecal samples were photometrically analysed for potassium, sodium, and calcium (flame photometry, Eppendorff EFOX 5033), with a chloridometer for chloride (Slamed Chloridmeter 50 μL), and photometrically for phosphate, using the modified vanadate molybdate method modified according to Gericke und Kurmies (1952) [57] (Thermo-Spectronic, Genesys 10 uv). Magnesium in feed, faeces, and urine was measured via spectrometry (Perkin Elmer Aanalyst 800) and a Weende analysis (VDLUFA 2012) was performed to quantify crude nutrients in feed and faecal samples [58]. Apparent digestibility (aD) was calculated using the following equation: aD (%) = (nutrient intake_feed_–nutrient output_faeces_)/nutrient intake_feed_. An ELISA kit (KAINOS Laboratories Inc., Tokyo, Japan) that was validated for feline samples was used to analyse serum samples for intact FGF23 [17]. Serum creatinine was photometrically measured at IDEXX Vet Med Laboratories GmbH, Ludwigsburg, Germany, and urine creatinine was analysed in-house (MicroVue Creatinine Assay Kit, Quidel Corporation).

### 2.5. Calculations and Statistical Analysis 

The apparent digestibility was calculated as follows: (intake–faecal excretion)/intake × 100. 

Delta calcium to phosphorus (∆Ca/P) in the faeces was calculated using the equation:

∆Ca/P = Ca/P_intake_ − Ca/P_faecal_

For statistical evaluation of results, a Student’s paired-t-test was performed to compare pre- and postprandial values and a t-test was used to compare values between groups. A Shapiro–Wilk test was performed to test for normality. Results with *p*-values ≤ 0.01, ≤0.001, and ≤0.05 were considered significantly different. 

## 3. Results

### 3.1. Nutrient Intake and Balance

Throughout the entire study, no adverse clinical effects were observed in any of the cats. Water intake significantly increased in the HNaCl group compared with the CON group (*p* ≤ 0.001; 41 ± 3 mL/kg vs. 29 ± 3 mL/kg BW/d), as well as urine volume (*p* ≤ 0.001; 22 ± 4 vs. 14 ± 3 g/kg BW/d). Dry matter intake decreased significantly (*p* ≤ 0.001), although apparent digestibility of dry matter was not affected by higher dietary concentrations of sodium chloride (Table 2). The apparent digestibility of phosphate, potassium, sodium, and chloride significantly increased (*p* = 0.028; *p* < 0.001; *p* < 0.001; *p* < 0.001), whereas the apparent digestibility of magnesium and calcium remained unaffected when table salt was added to the diet (Table 2). The addition of sodium chloride led to a highly significant increase in renal sodium and chloride excretion (*p* < 0.001). This also led to a significantly increased concentration of both elements in the urine (*p* < 0.001; Table 4), as well as increased retention of both elements (sodium: *p* = 0.001; chloride; *p* = 0.015). The retention of potassium and magnesium (*p* < 0.001; *p* = 0.008) was significantly increased in diet HNaCl as well. 

### 3.2. Blood Parameters 

**Figure 2:**
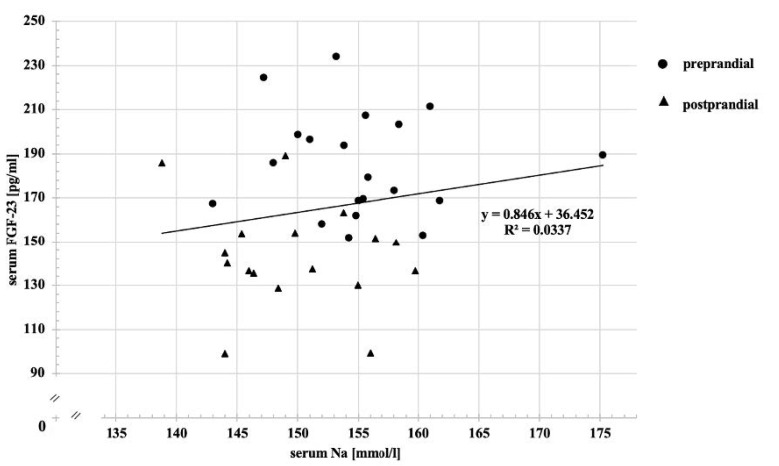
Correlation between serum Na and serum FGF23.

Pre-prandial serum sodium concentrations did not differ between groups (Table 3). Postprandially, the serum sodium concentration significantly decreased in the HNaCl group (*p* < 0.002). Values below the reference range were found in individuals at both time points in group HNaCl (1/11 pre- and 5/11 postprandially). In CON, the serum sodium concentrations were found to exceed the upper reference range in 3/11 cats pre- and 2/11 cats postprandially. In the HNaCl trial, this occurred in the pre-prandial serum samples of two cats. Serum calcium concentrations were significantly higher in group HNaCl before feeding (*p* = 0.018) but significantly decreased after food intake (*p* = 0.001), similar to the values in group CON (*p* = 0.013). Serum phosphate and FGF23 levels were only numerically changed by high sodium chloride intake. In alignment with unaffected serum FGF23 levels, no correlation was seen between serum FGF23 and sodium concentrations (Figure 2, adjusted for one statistical outlier). Comparing pre- and postprandial results, serum phosphate and FGF23 values significantly decreased 2 h after food intake in both groups (CON: *p* < 0.001, HNaCl *p* = 0.002). Due to lower postprandial serum phosphate concentrations, the serum calcium by phosphate product (sCaxP) significantly decreased in CON as well (*p* < 0.001). sCaxP values did not differ significantly between CON and HNaCl diets. In 5/11 cats from the HNaCl group and 2/11 cats from the CON group, the sCaxP values rose above the threshold of 55 mg/dl, given by Block et al. (2000) [59]. Serum potassium concentrations were significantly higher in group HNaCl at both time points (*p* < 0.001) compared with CON, with values above the reference range in 3/11 pre- and 2/11 postprandial samples. For serum creatinine concentrations, no effect of high salt intake was observed. In group CON, serum creatinine values significantly increased after food intake (*p* = 0.007) yet remained within the reference range. 

### 3.3. Urine Parameters

High sodium chloride intake led to a significantly higher urine volume (*p* < 0.001), with lower specific gravity (*p* < 0.001) and lower urinary creatinine concentrations (*p* < 0.001) compared with control feeding. Renal sodium excretion per kg BW increased ~4.5-fold and renal chloride excretion ~3.6-fold (Table 2) in trial HNaCl. Urine concentration of both minerals increased as well (*p* < 0.001; Table 4). Despite this increase in urine concentration combined with a ~50% higher urine volume, retention of sodium and chloride was significantly higher in the high salt group (*p* < 0.001, *p* < 0.015; Table 2). Urine concentration of potassium was significantly higher in group HNaCl (*p* < 0.001), despite a significant increase in potassium retention compared with CON (*p* < 0.001). Irrespective of the increase in urine volume, the urinary concentration of sodium, chloride, and potassium significantly increased in group HNaCl. Urine phosphate concentration significantly decreased (*p* = 0.003) after feeding HNaCl. However, there was no difference between the groups when corrected for creatinine (P/Crea).

## 4. Discussion

This study investigated the effects of high sodium chloride intake on serum FGF23 and parameters of mineral balance. The purpose of the study was to gather knowledge regarding implications on calcium and phosphate metabolism, blood pressure, and renal health in cats. Therefore, we aimed to produce a test and a control diet with identical concentrations of nutrients, apart from sodium and chloride. The cation–anion balance of both diets was adjusted by adding potassium to the HNaCl diet to prevent effects on electrolyte homeostasis, e.g., via its impact on renal excretion of certain minerals. To limit strain on the cats, blood samples were only drawn at one postprandial time point in this study. We cannot exclude the possibility that the concentrations of serum FGF23 and other parameters were influenced by the sodium chloride intake at different time points after food intake. Nonetheless, comparison of pre- and postprandial serum values in cats fed a high sodium or control diet gave insight to the potential effects of high sodium chloride intake on nutrient balance and selected serum parameters. 

Studies in dogs [54] and cats [61] showed that complete and balanced diets tended to lower serum FGF23 concentrations 3 h postprandially. The same was seen in this study, independent of the sodium chloride content of the diet: both groups showed a significant decrease in postprandially measured serum FGF23. No differences were detected between the feeding groups. In contrast to observations made in mice and humans [24,32], high intake of sodium chloride for a moderate time span (28 days) did not cause a decrease in serum FGF23 concentrations in cats, neither in the fasted state nor postprandially. However, these results are consistent with studies demonstrating that high sodium chloride intake neither caused changes in blood pressure nor in serum aldosterone levels of healthy cats [51]. Humans and dogs tend to show a higher prevalence of increased blood pressure compared with cats; for example, only 69% [47] of feline patients with CKD were found to be hypertensive, compared with about 86% of humans [62] and up to 93% of dogs [63,64] developing increased blood pressure with renal failure.

The significant decrease of postprandial serum sodium values 3 h after food intake was probably caused by the highly significant increase observed in renal excretion of sodium, combined with the highly significant increase in water consumption. A possible explanation for this assumed correlation is that the high sodium chloride intake led to an upregulation of renal sodium excretion with a transient drop in serum sodium concentrations. In addition to sodium chloride, the potassium supply, and therefore its serum concentrations, might have affected serum FGF23 concentrations [65]. Specifically, a study in humans found a potential connection between low dietary potassium intake and an increase in FGF23 [66]. Therefore, it was expected that a high sodium chloride supply combined with a high potassium intake (as in group HNaCl) would cause a decrease in serum FGF23. This hypothesis was also supported by findings from Yeung et al. (2021), who observed an inverse correlation between renal potassium excretion and serum FGF23 in humans [67]. Despite a higher potassium and sodium supply, plus increased renal potassium excretion in the HNaCl group, no effect on serum FGF23 values was seen in cats. In this respect, cats appear to hold a divergent position compared with other monogastric species. 

Next to FGF23, phosphate metabolism is also influenced by sodium intake in humans. The urinary excretion of sodium and phosphorus were found to correlate strongly in healthy adults [68]. In our study with cats, despite a decrease in the urinary phosphate concentration in group HNaCl, there was no impact on renal phosphate excretion per kg BW or the urine phosphate to creatinine ratio. The drop in urinary phosphate concentration could be explained by the increased diuresis from significantly higher urine volume with high sodium chloride intake. Similarly, serum phosphate remained unaffected by higher dietary sodium chloride intake. However, the apparent digestibility of phosphate was increased in group HNaCl. Weak, but positive correlations were found (chloride R^2^ = 0.284 > NaCl R^2^ = 0.259 > sodium R^2^ = 0.194) when testing the correlation between apparent digestibility of phosphorus, along with the intake of either sodium and chloride alone, or the sodium chloride compound, respectively. Similar effects were found in pigs [69] and rats [70], where adding sodium chloride to the diet also led to an increase in apparent digestibility of phosphorus. 

When evaluating apparent phosphorus digestibility, its source also needs to be taken into consideration. As shown in cats, highly soluble inorganic phosphates can have a higher availability in the gut compared with organic sources, resulting in an increase in apparent phosphorus digestibility [71]. Our study demonstrated that the source and amount of phosphate did not differ between the groups. The difference in apparent phosphorus digestibility was probably caused by the sodium chloride concentration of the diet, which was possibly mediated by the Na-/P-co-transporter in the gut, as demonstrated in rats [70].

Furthermore, the faecal calcium to phosphorus ratio (Ca/P_faecal_) was significantly higher in the HNaCl group compared with CON (3.6 ± 0.5; 3.0 ± 0.4; *p* = 0.008). Consequently, ∆Ca/P was significantly lower when sodium chloride was added to the diet (CON: −1.7 ± 0.4; HNaCl: −2.3 ± 0.5; *p* = 0.007). The wider Ca/P_faecal_ ratio and decrease in ∆Ca/P imply that even though the source of phosphate did not differ between groups, more phosphate was digested in the HNaCl group. These results differ from previous findings by Böswald et al. (2020), who found that highly soluble phosphates led to significantly lower ∆Ca/P values in dogs [72] but not in cats [73]. A possible explanation for the higher digestibility of phosphorus in our study could be from an increase in phosphate availability due to the high sodium chloride intake. The combination of high sodium chloride intake with a high phosphate supply, especially from highly soluble sources, could further increase the apparent digestibility of phosphate. Our results suggest that excess dietary phosphorus, which is often found in commercial pet food [74], combined with high sodium chloride, may increase the phosphorus burden on the body, potentially causing adverse health effects, as described in cats and other species [20,21,22,23]. Consequently, the addition of sodium chloride to increase urine volume and dilute urinary phosphorus concentration, to reduce potential adverse effects of phosphate, may be counterproductive. Nguyen et al. (2017) demonstrated that the addition of sodium significantly above levels recommended by the NRC (40 mg/MJ ME) or FEDIAF (45 mg/MJ ME) is common practice in commercial diets for cats. In the 26 products investigated, sodium content ranged from 110 mg/MJ ME in a renal diet up to 820 mg/MJ ME in a diet formulated for urolithiasis, which exceeds the safe upper limit suggested by Nguyen et al. (740 mg/MJ ME) [75]. The results of a study on rats underlined this potential issue. Specifically, feeding a high sodium chloride diet after recovery from an acute kidney injury caused accelerated tubulointerstitial damage, increased hypertension, and albuminuria, whereas parameters continued to be stable when fed a low sodium chloride diet [76]. Furthermore, lower sodium retention and blood pressure was found in FGF23-deficient mice [24]. Therefore, low FGF23 serum levels and low sodium chloride intake may have a protective effect for hypertension in some species. In contrast, no effect of sodium chloride intake on FGF23 serum levels was measured in the cats of our study. This matched previous results that found blood pressure remained unaffected by adding sodium chloride to the diet of this species [51]. These results imply differences in the correlation between FGF23 and dietary sodium chloride in cats compared with other species. This calls for further research to investigate regulatory mechanisms of FGF23 in felines. FGF23 reduces the production of vitamin D, which leads to an activation of RAAS. Chronically increased FGF23 concentrations indirectly lower αKlotho values [12], potentially elevating FGF23 serum concentrations even more. This further justifies continued research on the connection between dietary sodium chloride and phosphorus metabolism in cats, especially with the knowledge that elevated FGF23 serum concentration is an early marker for kidney damage in felines and several other species [15,16,17,18,19]. 

## 5. Conclusions

In the present study on cats, high sodium chloride intake did not affect serum FGF23 concentrations as observed in other species. Here, serum FGF23 and sodium chloride intake were shown to be unrelated. This confirms a different relationship between FGF23 and sodium chloride in cats compared with other species, limiting possible conclusions regarding blood pressure and other clinical implications. The impact of sodium chloride supply on the apparent digestibility of phosphorus should be considered, as it could lead to potential adverse health effects. This is especially important due to the high supply of these elements in many complete cat food products on the market. To establish a safe upper limit for sodium chloride in cats, it should be considered that excessive sodium chloride intake may have different adverse health effects compared with other species. Furthermore, the correlation between sodium chloride supply and phosphorus digestibility must be considered.

## Figures and Tables

**Table 1:** Composition of diets.

		CON	HNaCl
Ingredients basal diet	%	Beef (Heart, Steak) 72 Rice 24 Cellulose 1 Rapeseed oil 3	
DM	g/kg	431	
GE	MJ/kg DM	27	
Crude protein	g/kg DM	439	
Crude fat		362	
Crude fibre		35	
Crude ash		16	
NfE		77	
Ca		4.6 ± 0.2	
P		3.5 ± 0.1	
K		8	18
Mg		0.75 ± 0.2	
Na		1.4	12.1
Cl		6.1	29.1
Ca/P	-	1.3/1	
Vit. D_3_	IU/kg DM	428 ± 30	

DM = dry matter, GE = gross energy, NfE = nitrogen-free extract, Ca = calcium, P = phosphate, K = potassium, Mg = magnesium, Na = sodium, Cl = chloride, Ca/P = calcium–phosphorus ratio.

**Table 2:** Intake, renal and faecal excretion, retention per kg body weight and day, and apparent digestibility [%] of dry matter and minerals (mean ± standard deviation).

Nutrient	Diet	Intake	Faecal Excretion	aD	Renal Excretion	Retention
DM [g]	CON	13 ± 2	1.3 ± 0.3	90 ± 2	-	-
	HNaCl	12 ± 1 ***	1.2 ± 0.5	90 ± 3	-	-
Ca [mg]	CON	64 ± 4	58 ± 11	10 ± 17	0.4 ± 0.1	6 ± 11
	HNaCl	55 ± 4 ***	47 ± 16 *	16 ± 26	0.4 ± 0.1	8 ± 14
P [mg]	CON	49 ± 3	19 ± 4	60 ± 9	14 ± 5	15 ± 4
	HNaCl	42 ± 3 ***	13 ± 4 **	69 ± 10 *	16 ± 4	13 ± 4
K [mg]	CON	107 ± 6	3 ± 1	97 ± 1	92 ± 14	12 ± 11
	HNaCl	218 ± 14 ***	2 ± 1	99 ± 1 ***	174 ± 32 ***	42 ± 21 ***
Mg [mg]	CON	12 ± 1	6 ± 1	46 ± 10	2 ± 1	4 ± 2
	HNaCl	8 ± 1 ***	5 ± 1 **	43 ± 17	1 ± 1	2 ± 1**
Na [mg]	CON	19 ± 1	3 ± 1	86 ± 5	29 ± 4	−12 ± 4
	HNaCl	149 ± 10 ***	3 ± 1	98 ± 1 ***	136 ± 26 ***	10 ± 19 ***
Cl [mg]	CON	83 ± 5	3 ± 1	96 ± 2	94 ± 12	−14 ± 10
	HNaCl	376 ± 52 ***	3 ± 1	99 ± 0 ***	346 ± 67 ***	27 ± 71*

aD = apparent digestibility, DM = dry matter, Ca = calcium, P = phosphate, K = potassium, Mg = magnesium, Na = sodium, Cl = chloride, * *p* ≤ 0.05, ** *p* ≤ 0.01, *** *p* ≤ 0.001 between groups.

**Table 3:** Concentrations of serum parameters after 28 days of high NaCl or control feeding in cats (mean ± standard deviation).

Serum	Time Point	CON	HNaCl	Reference Range
FGF23 [pg/mL]	Pre-prandial	202 ± 53	183 ± 23	-
	Postprandial	142 ± 22 ^#^	146 ± 25 ^#^	
Creatinine [mmol/L]	Pre-prandial	0.14 ± 0.01	0.14 ± 0.02	0.08–0.2
	Postprandial	0.15 ± 0.01 ^#^	0.15 ± 0.02	
Ca [mmol/L]	Pre-prandial	2.3 ± 0.1	2.5 ± 0.2 **	2.2–2.9
	Postprandial	2.2 ± 0.2 ^#^	2.3 ± 0.1 ^#^	
P [mmol/L]	Pre-prandial	1.8 ± 0.2	1.9 ± 0.3	0.8–2.2
	Postprandial	1.4 ± 0.1 ^#^	1.6 ± 0.2 ^#^	
sCaxP [mg^2^/dL^2^]	Pre-prandial	52 ± 6	60 ± 10	<55/<70 ^Δ^
	Postprandial	39 ± 3 ^#^	44 ± 8 ^#^	
K [mmol/L]	Pre-prandial	4.1 ± 0.3	5.7 ± 1.0 ***	3.3–5.8
	Postprandial	4.0 ± 0.1	5.4 ± 0.5 ***	
Na [mmol/L]	Pre-prandial	156 ± 4	154 ± 9	147–159
	Postprandial	157 ± 2	146 ± 4 ^#^***	

Ca = calcium, P = phosphate, sCaxP = serum calcium by phosphorus product, K = potassium, Na = sodium, ** *p* ≤ 0.01, *** *p* ≤ 0.001 between groups, ^#^ pre- and postprandial values within one group significantly differ (*p* < 0.05), Δ [59,60].

**Table 4:** Concentrations of urine parameters after 28 days of high NaCl or control feeding in cats (mean ± standard deviation).

Urine	CON	HNaCl	Reference Range
Creatinine [mmol/L]	32 ± 4	20 ± 2 ***	-
USG [mg/mL]	1060 ± 2	1052 ± 5 ***	1001–1085
Volume [mL/kg BW/d]	14 ± 3	22 ± 4 ***	<50
Na [g/L]	2.0 ± 0.3	6.2 ± 0.5 ***	-
Cl [g/L]	6.6 ± 0.7	15.8 ± 1.3 ***	-
K [g/L]	6.5 ± 0.7	8.0 ± 0.6 ***	-
P [g/L]	1.0 ± 0.2	0.7 ± 0.1 **	-
P/Crea [mmol/L]	1.0 ± 0.3	1.2 ± 0.2	-

USG = urine specific gravity, Na = sodium, Cl = chloride, K = potassium, P = phosphorus, P/Crea = phosphorus–creatinine ratio, ** *p* ≤ 0.01, *** *p* ≤ 0.001 between groups.

## Data Availability

Data presented in this study are available on request from the corresponding author.
